# Unraveling allosteric signaling of G protein-coupled receptors (GPCRs) by single-molecule fluorescence

**DOI:** 10.1007/s12551-025-01361-3

**Published:** 2025-08-27

**Authors:** Michael Tope Agbadaola, Daniel Hilger, Sandro Keller, Georg Krainer

**Affiliations:** 1 Department of Chemical Sciences, Dominion University, Ibadan, Nigeria; 2 Biophysics, Institute of Molecular Biosciences (IMB), University of Graz, NAWI Graz, Graz, Austria; 3 Field of Excellence BioHealth, University of Graz, Graz, Austria; 4 BioTechMed-Graz, Graz, Austria; 5 Department of Pharmaceutical Chemistry, Marburg University, Marburg, Germany

**Keywords:** G protein-coupled receptors, Single-molecule Förster resonance energy transfer, Membrane receptors, Single-molecule fluorescence

## Abstract

G protein-coupled receptors (GPCRs) are the largest and most functionally diverse family of membrane receptors in eukaryotes. They play central roles in numerous physiological processes and are implicated in a wide range of diseases, making them prime targets for therapeutic intervention. Allostery is central to GPCR function, enabling the transmission of extracellular signals across the membrane into intracellular responses. Specifically, three key allosteric phenomena—ligand efficacy, biased signaling, and allosteric modulation—are fundamental to GPCR signaling and have been explored through various approaches. In this review, we summarize how single-molecule fluorescence techniques, particularly single-molecule Förster resonance energy transfer (smFRET) and single-molecule photoisomerization-related/protein-induced fluorescence enhancement (smPIFE), have deepened our understanding of these allosteric processes. We discuss existing gaps in our understanding of GPCR allostery and how these techniques could be leveraged to address these challenges, driving the development and design of more effective and selective therapeutics.

## Introduction

G protein-coupled receptors (GPCRs) are the largest and clinically most important family of membrane receptors in eukaryotes (Lagerström & Schiöth 2008; Lorente et al. 2025). They regulate a variety of intracellular signaling processes in response to a wide array of external signals, including photons, ions, small organic molecules, peptides, proteins, lipids, and sugars (Agyemang et al. 2024; Hilger et al. 2018; Quast & Margeat 2019). GPCRs are categorized according to their structural and functional characteristics into Class A (rhodopsin-like family), Class B (secretin and adhesion family), Class C (glutamate family), and Class F (frizzled family) (Bjarnadóttir et al. 2006; Hu et al. 2017; Stevens et al. 2013). Due to their diverse roles in key cellular processes, GPCRs are implicated in numerous diseases, including cardiovascular, psychiatric, and metabolic disorders, as well as cancer, making them prominent drug targets, with around 35% of therapeutic drugs acting on these receptors (Boczek et al. 2021; Lappano & Maggiolini 2011, 2017; Morris & Malbon 1999; Overington et al. 2006; Weis & Kobilka 2018; Zhao et al. 2024; Lorente et al. 2025).

GPCRs transmit signals across the cell membrane through allosteric communication, relaying information from extracellular signals to various intracellular transducers (Bock & Bermudez 2021; Hilger et al. 2018; Murthy et al. 2021; Smith et al. 2018; Weis & Kobilka 2018). The mechanisms underlying this allosteric communication have become increasingly well-defined, revealing a complex interplay of structural and functional changes within the receptor (Quast & Margeat 2019). Understanding these complex mechanisms has been crucial for designing therapeutics that are selective—preferentially targeting a particular receptor conformation and, thus, signaling pathway—or even specific—exclusively engaging the desired conformation or pathway (Conflitti et al. 2025).

Key allosteric phenomena—including ligand efficacy, biased signaling, and allosteric modulation—govern GPCR activation and downstream signaling pathways. These processes have been explored using approaches ranging from pharmacology (Eiger et al. 2022; Seyedabadi et al. 2019; Urban et al. 2007; Yang et al. 2021) to biophysics (Gregorio et al. 2017; Grime et al. 2020; Lamichhane et al. 2015; Namkung et al. 2016; Ploetz et al. 2016; Wingler et al. 2019), which have provided important insights into structure–function relationships (Bruzzese et al. 2020; Rasmussen et al. 2011; Zhang et al. 2017), signaling specificity (Asher et al. 2022; Lamichhane et al. 2020), and the development of targeted therapeutic agents (Lappano & Maggiolini 2011 and 2017; Lindberg et al. 2005). Among these approaches, single-molecule fluorescence methods have emerged as a cornerstone for investigating GPCR signaling phenomena, offering unprecedented insights that deepen our understanding of allosteric signaling in GPCRs (Agam et al. 2023; Agyemang et al. 2024; Quast & Margeat 2019).

In this review, we discuss recent advancements in our understanding of allosteric signaling phenomena in GPCRs through single-molecule fluorescence approaches. We begin with an overview of the key allosteric phenomena in GPCRs—ligand efficacy, biased signaling, and allosteric modulation—and introduce the main single-molecule fluorescence methods used to study GPCR allostery. We describe how single-molecule Förster resonance energy transfer (smFRET) and single-molecule photoisomerization-related/protein-induced fluorescence enhancement (smPIFE) have advanced our understanding of the mechanisms underlying these allosteric phenomena. We conclude with a critical discussion of these findings and an outlook on future directions for advancing our understanding of GPCR allostery.

## Allosteric phenomena

The mechanisms linking stimuli from extracellular ligands to intracellular transducers that activate GPCRs are complex. In its simplest form, an extracellular ligand binds to a so-called orthosteric binding site on the GPCR, triggering conformational changes within the receptor’s structural core, typically in its seven transmembrane domains (7TMD), but also in other domains, such as various extracellular domains (ECDs) present in Class B, C, and F GPCRs, which have been observed to be particularly important in ligand binding (Liu et al. 2023; Olofsson et al. 2014; Schulte 2024; Tora et al. 2018). This enables the receptor to interact with and transmit signals to intracellular transducers, such as heterotrimeric G proteins, which consist of α, β, and γ subunits (G_αβγ_). Upon GPCR binding, the G protein undergoes nucleotide exchange at its α-subunit, leading to dissociation from the G protein complex and activation of second-messenger pathways that drive various physiological responses within the cell (Mafi et al. 2022; Weis & Kobilka 2018).

An alternative signaling pathway involves the recruitment of other transducers, like arrestins and kinases. Arrestins are typically recruited after the receptor’s C-terminal tail is phosphorylated by G protein-coupled receptor kinases (GRKs). Once bound, arrestins inhibit G protein signaling, thereby desensitizing the receptor (Benovic 2021; Chen & Tesmer 2022; Hilger et al. 2018; Ranjan et al. 2017; Weis & Kobilka 2018; Wess et al. 2023). This intricate interplay between extracellular ligand binding, receptor conformational changes, and intracellular transducer coupling forms the basis of the complex activation and signaling mechanisms of GPCRs. In the following, we briefly introduce three of the most important allosteric phenomena observed for GPCRs, namely, ligand efficacy, biased signaling, and allosteric modulation.

### Ligand efficacy

**Fig. 1:**
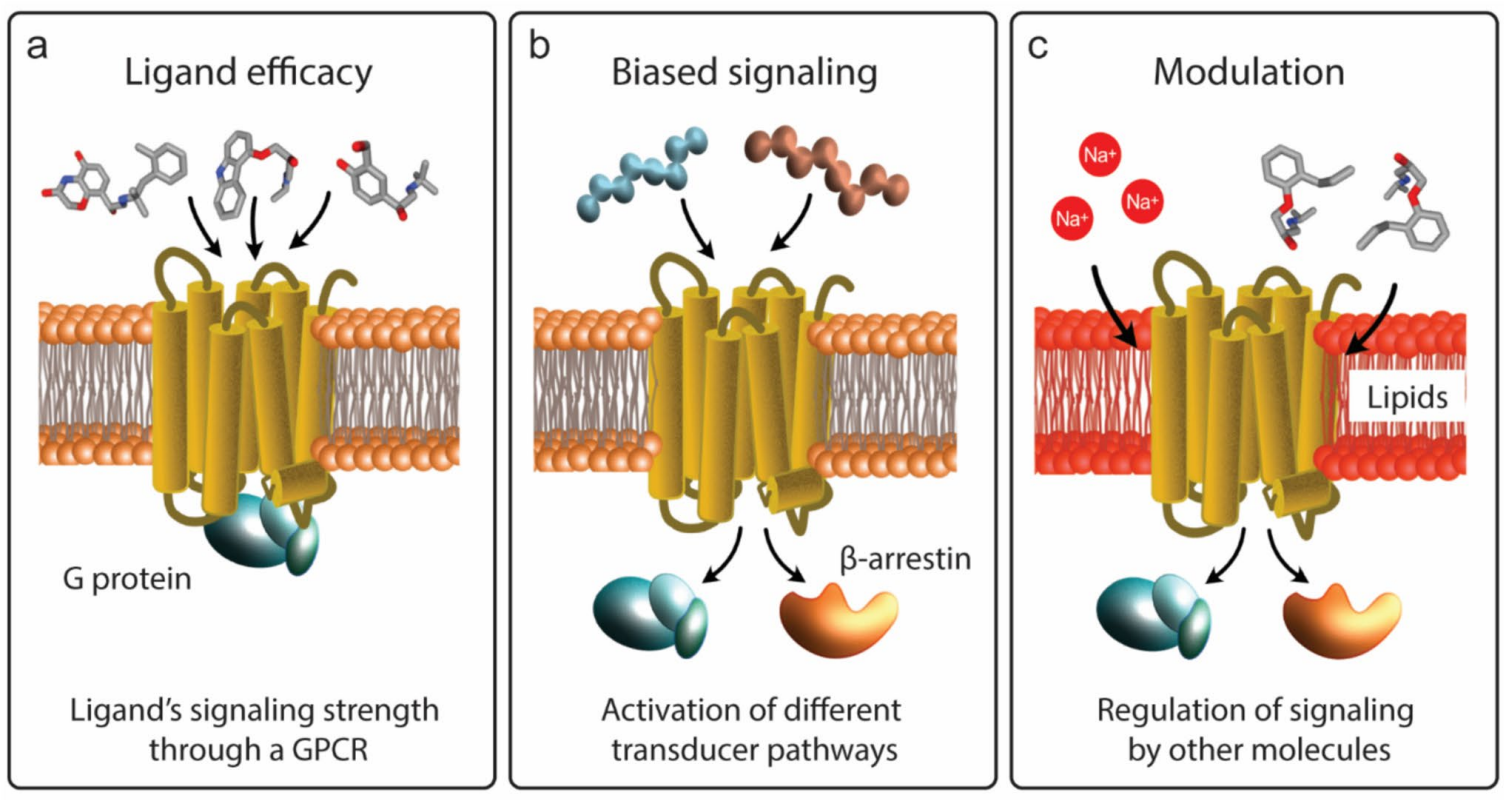
Overview of GPCR allosteric phenomena. The allosteric signaling phenomena, ligand efficacy, allosteric modulation, and biased signaling, are important processes in GPCR signaling. (**a**) GPCRs respond to ligands to an extent that corresponds to their respective efficacies. (**b**) Biased ligands can recruit specific transducers, leading to the activation of a specific signaling pathway at the expense of the others. (a) Allosteric modulators increase or decrease the efficacy and/or affinity of ligands, thereby modulating their receptor activation patterns

The degree to which a ligand can induce activation in a receptor, resulting in a measurable physiological response, is called ligand efficacy (Neubig et al. 2003) (Fig. 1a). Based on efficacy, ligands are classified into four main types: full agonists, partial agonists, neutral antagonists, and inverse agonists (Kenakin 2002; Wacker et al. 2017). Full agonists promote maximal activation by stabilizing the active conformation of receptors. Partial agonists, in contrast, trigger only intermediate activation, leading to a reduced signaling outcome. Neutral antagonists bind to the same site as agonists but do not affect the receptor activity, while inverse agonists reduce the receptor’s basal activity, counteracting any constitutive receptor signaling. Understanding the underlying ligand-induced conformational changes of a receptor is, therefore, crucial for designing therapeutics that can precisely modulate receptor states to achieve desired physiological outcomes.

### Biased signaling and functional selectivity

While agonists generally promote transducer coupling to the ICD of GPCRs, the recruitment efficiency of specific transducers can vary among different ligands for the same receptor. Agonists that recruit both G proteins and arrestins to the same extent are referred to as balanced agonists (Rajagopal et al. 2010). However, some ligands selectively activate a particular signaling pathway while minimizing others, a phenomenon known as biased signaling (Fig. 1b) or functional selectivity (Correll & McKittrick 2014; Kenakin 2013; Kolb et al. 2022; Smith et al. 2018; Wisler et al. 2014). Biased agonists act by inducing distinct conformational changes in the receptor, differing from those triggered by balanced agonists. Understanding the conformational “signature” of biased ligands is crucial for designing GPCR-based therapeutics, as it enables the selective targeting of desired pathways, reducing potential side effects caused by the activation of alternative pathways (Smith et al. 2018; Wootten et al. 2018).

### Allosteric modulation

Allosteric modulation (Fig. 1c), the process by which a binding event at one site of a protein affects binding or activity at other sites, is an important feature of protein regulation in cells. The affinity and signaling efficacy of orthosteric ligands, whether biased or balanced, can be influenced by various cellular components or exogenous agents known as allosteric modulators. These modulators, including small molecules, ions, peptides, or lipids, bind to allosteric sites on the receptor that are structurally distinct from the orthosteric binding site but, nevertheless, modulate the receptor’s response to orthosteric ligands (Foster & Conn 2017). Allosteric modulators are classified based on how they modulate orthosteric ligand activity. Positive allosteric modulators (PAMs) enhance the response to orthosteric agonists, thereby amplifying their physiological activity (Foster & Conn 2017). PAMs may increase agonist efficacy, partially activate receptors, or even bias the receptor toward specific signaling pathways (Christopoulos 2014; Makita et al. 2007; Rook et al. 2013). In contrast, negative allosteric modulators (NAMs) reduce agonist effects by decreasing agonist binding, signaling efficacy, or both. PAMs and NAMs function by shifting the equilibrium between pre-existing conformational states (Cao et al. 2021); in other words, binding of these effectors leads to the stabilization or destabilization of particular receptor conformations (Kumar et al. 2023; Kruse et al. 2013; Liu et al. 2019; Shaye et al. 2020; Srivastava et al. 2014). Allosteric modulators represent a promising class of therapeutic agents for precisely modulating GPCR responses with high specificity, and some have already reached the market (Shen et al. 2023). Understanding the mechanisms by which these modulators influence receptor activity is essential for developing highly specific and selective therapeutics.

### Approaches to studying allosteric phenomena

Ligand efficacy, biased signaling, and allosteric modulation are key pharmacological concepts that impact GPCR activation and signaling, making it essential to understand the mechanisms underlying these processes. Significant efforts have been made to uncover mechanistic insights into these complex allosteric behaviors using a range of techniques, and several approaches exist for investigating GPCR allosteric phenomena.

Structural snapshots of GPCRs in distinct conformations—bound to orthosteric ligands in the presence or absence of allosteric modulators alone or in complex with transducers or conformational state-stabilizing antibody fragments—have been obtained through X-ray crystallography (Che et al. 2018; Cherezov et al. 2007; Jaakola et al. 2008; Kang et al. 2015; Kruse et al. 2013; Liu et al. 2019; Manglik et al. 2012; McCorvy et al. 2018; Rasmussen et al. 2011; Srivastava et al. 2014; Tsai et al. 2018) and cryogenic electron microscopy (Bueno et al. 2020; Gao et al. 2021; García-Nafría et al. 2018; Kato et al. 2019; Lee et al. 2020; Shaye et al. 2020; Zhang et al. 2017). These approaches have been instrumental in revealing over 1700 structures of 238 unique GPCRs to date (Herrera et al. 2025).

Beyond structures, techniques such as double electron–electron resonance (DEER) spectroscopy (Wingler et al. 2019; H. Zhang et al. 2018; Zhao et al. 2024), nuclear magnetic resonance (NMR) spectroscopy (Bumbak et al. 2023; Casiraghi et al. 2019; Frei et al. 2020; Huang et al. 2021; Imai et al. 2020; Isogai et al. 2016; Liu et al. 2012; Sušac et al. 2018; Wu et al. 2020; Ye et al. 2016), and molecular dynamics (MD) simulations (Aranda-García et al. 2025; Bansal et al. 2023; Dror et al. 2011; Huber et al. 2004; Nygaard et al. 2013) have provided crucial insights into GPCR conformational dynamics and heterogeneity. Techniques such as hydrogen–deuterium exchange mass spectrometry (HDX-MS) (Josephs et al. 2021; Kumar et al. 2023; Latorraca et al. 2025; Qiu et al. 2024; Shukla et al. 2014; Toporowska et al. 2024; L. Yang et al. 2015) and cross-linking mass spectrometry (XL-MS) (Xia et al. 2020; Yen et al. 2017; Yuan et al. 2023) have further contributed by mapping conformational states and protein–protein interactions, thereby enriching our understanding of GPCR structural dynamics and allosteric modulation.

Over the past decade, single-molecule techniques (Fernandes et al. 2021; Gregorio et al. 2017; Habrian et al. 2019; Lamichhane et al. 2015, 2020; Liauw et al. 2022; Schafer et al. 2025; Wei et al. 2022) have provided crucial insights into multi-state conformational heterogeneity, oligomerization, and inter-state dynamic transitions that are central to allosteric processes. These methods are particularly valuable for observing individual GPCR molecules, offering high-resolution insights into GPCR conformational dynamics at the molecular level. In the following section, we review single-molecule fluorescence approaches for studying GPCR signaling.

## Single-molecule fluorescence approaches for studying GPCRs

GPCRs are highly dynamic proteins that can adopt various conformations in response to external stimuli. Previously believed to switch between only two conformations, active and inactive states, recent advances have revealed that many GPCRs undergo multi-state transitions. This implies that, rather than functioning as simple on/off switches, GPCRs explore a range of conformational states, with transitions occurring among all states involved (Hilger 2021; Manglik & Kobilka 2014; Quast & Margeat 2019).

Single-molecule approaches, particularly single-molecule fluorescence, have been instrumental in expanding our understanding of GPCR signaling by allowing observation of individual receptor molecules in complex membrane environments, offering high-resolution insights into their dynamic conformational behaviors. Single-molecule fluorescence can reveal heterogeneous conformational states (Cao et al. 2021; Fernandes et al. 2021; Gregorio et al. 2017; Lamichhane et al. 2015), rare events (Olofsson et al. 2014), and non-uniform kinetics (Liu et al. 2023; Zhao et al. 2024) pivotal to GPCR activation and allosteric signaling, which are often missed in ensemble-averaged experiments. In the following, we describe single-molecule Förster resonance energy transfer (smFRET) and single-molecule photoisomerization-related/protein-induced fluorescence enhancement (smPIFE), two highly important single-molecule fluorescence techniques that have proven especially valuable for studying conformational states and dynamics critical for GPCR allosteric signaling.

### smFRET

**Fig. 2:**
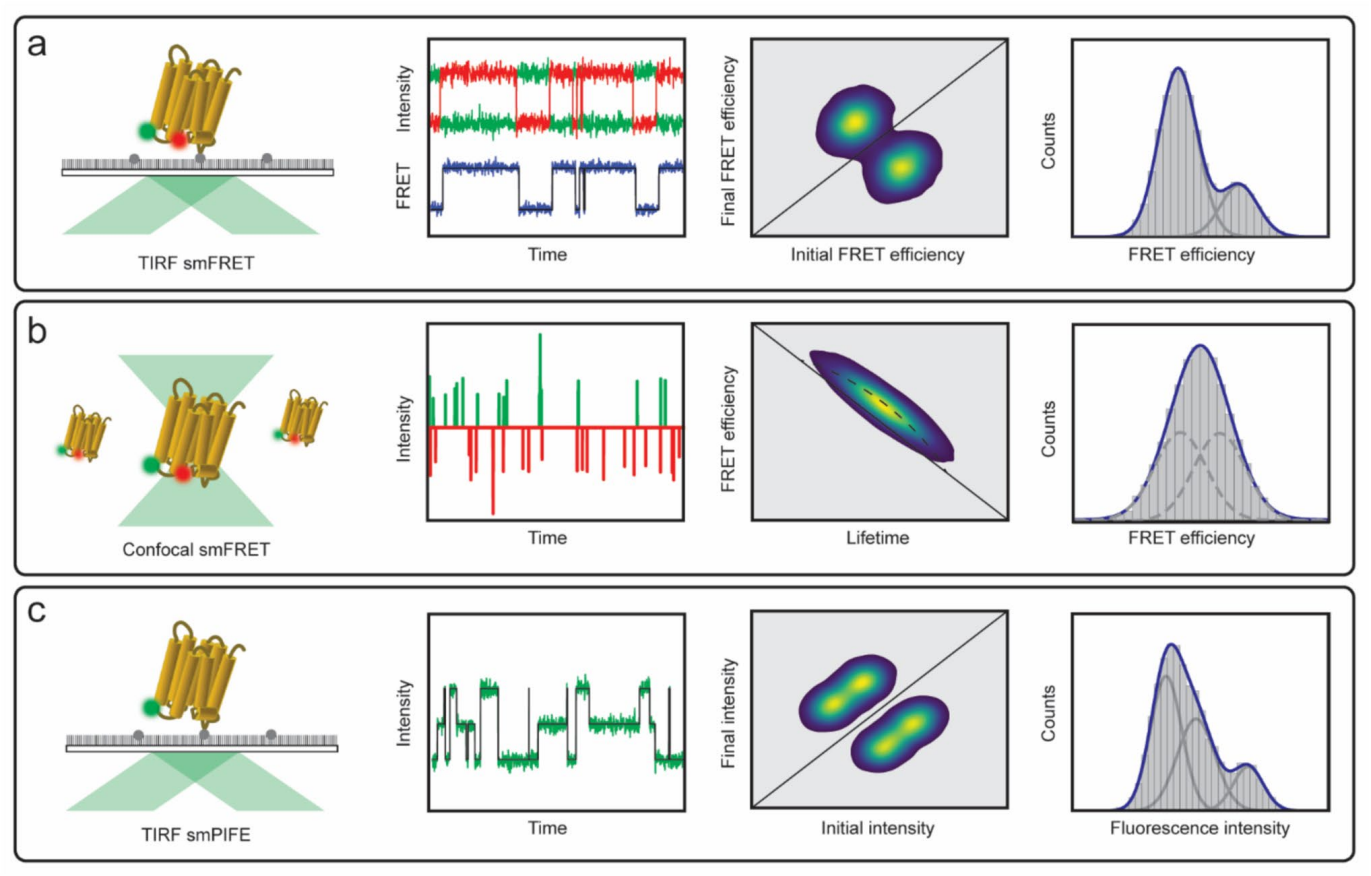
Single-molecule fluorescence approaches for studying GPCR allostery. (**a**) TIRF smFRET. Receptor labeled with a donor (green) and acceptor (red) fluorescent pair is immobilized on a surface and monitored on a TIRF microscope. smFRET trajectories of the fluorophores are recorded over time and analyzed to obtain information on the conformational states and kinetics. (**b**) Confocal smFRET. Receptor labeled with a donor (green) and acceptor (red) fluorescent pair is allowed to freely diffuse through a confocal volume and monitored on a confocal microscope. smFRET burst time traces of the fluorophores are recorded over time and analyzed to obtain information on the conformational states and kinetics. (**c**) TIRF smPIFE. Receptor labeled with a single fluorophore (green) is immobilized on a surface and monitored on a TIRF microscope. smPIFE trajectories are acquired from the fluorescent intensities of the fluorophore over time and analyzed to obtain information on the conformational states and dwell time

smFRET provides detailed insights into receptor dynamics by measuring the distance-dependent energy transfer between fluorophores attached to a protein (Krainer et al. 2019; Lerner et al. 2018). In this technique, a donor fluorophore transfers excitation energy non-radiatively to an acceptor fluorophore, with the energy transfer efficiency (*E*) depending on the fluorophores’ distance, orientation, and spectral overlap. For conventional fluorophore pairs, the Förster radius is typically around 5 nm, allowing distance measurements in the range of approximately 2 to 11 nm (Voithenberg and Lamb  2018). In a standard smFRET experiment, the target GPCR is labeled with a donor–acceptor fluorophore pair and is either immobilized on a surface for monitoring with total internal reflection fluorescence (TIRF) microscopy or allowed to diffuse through an observation volume and monitored with a confocal microscope. In TIRF-based smFRET, conformational changes of the GPCR are tracked through FRET efficiency trajectories, which reflect changes in the distance between the two fluorophores (Fig. 2a). These trajectories are then analyzed using statistical models to identify conformational states and the kinetics of transitions between them. To gauge the distributions of conformational states, FRET values within each single-molecule trajectory are binned into a FRET efficiency histogram. TIRF-based smFRET experiments are typically suited for probing timescales ranging from milliseconds to seconds. In confocal smFRET on freely diffusing molecules, fluorescence bursts are detected for each single molecule observed (Fig. 2b). From these bursts, fluorescence intensities, and, depending on the detection scheme, fluorescence lifetimes and anisotropies, can be derived. These parameters allow the calculation of FRET efficiencies and accumulation of single-molecule events in FRET efficiency histograms. These can be used to analyze conformations and dynamics on timescales from nanoseconds to seconds. The underlying principles and methodological considerations of smFRET have been comprehensively reviewed elsewhere (Agyemang et al. 2024; Hellenkamp et al. 2018; Lakowicz 2006; Modak et al. 2024; Quast & Margeat 2019; Tian et al. 2017).

### smPIFE

smPIFE uses a different approach, with GPCRs labeled with a single fluorophore whose fluorescence properties change in response to environmental shifts (Lamichhane et al. 2015, 2020; Maeda et al. 2014; Stennett et al. 2015; Wei et al. 2022). Rather than monitoring energy transfer between two probes, smPIFE detects photoisomerization of a single fluorophore based on environmental changes around the probe. Conformational changes of the GPCR are recorded as fluorescence intensity trajectories, typically using TIRF microscopy, reflecting shifts in the local environment of the fluorophore (Fig. 2c). These intensity changes can be analyzed using statistical models to define the conformational states and the kinetics of transitions between them. More technical insights into smPIFE can be found in elsewhere (Luo et al. 2007; Markiewicz et al. 2012; Myong et al. 2009).

### Labeling

**Fig. 3:**
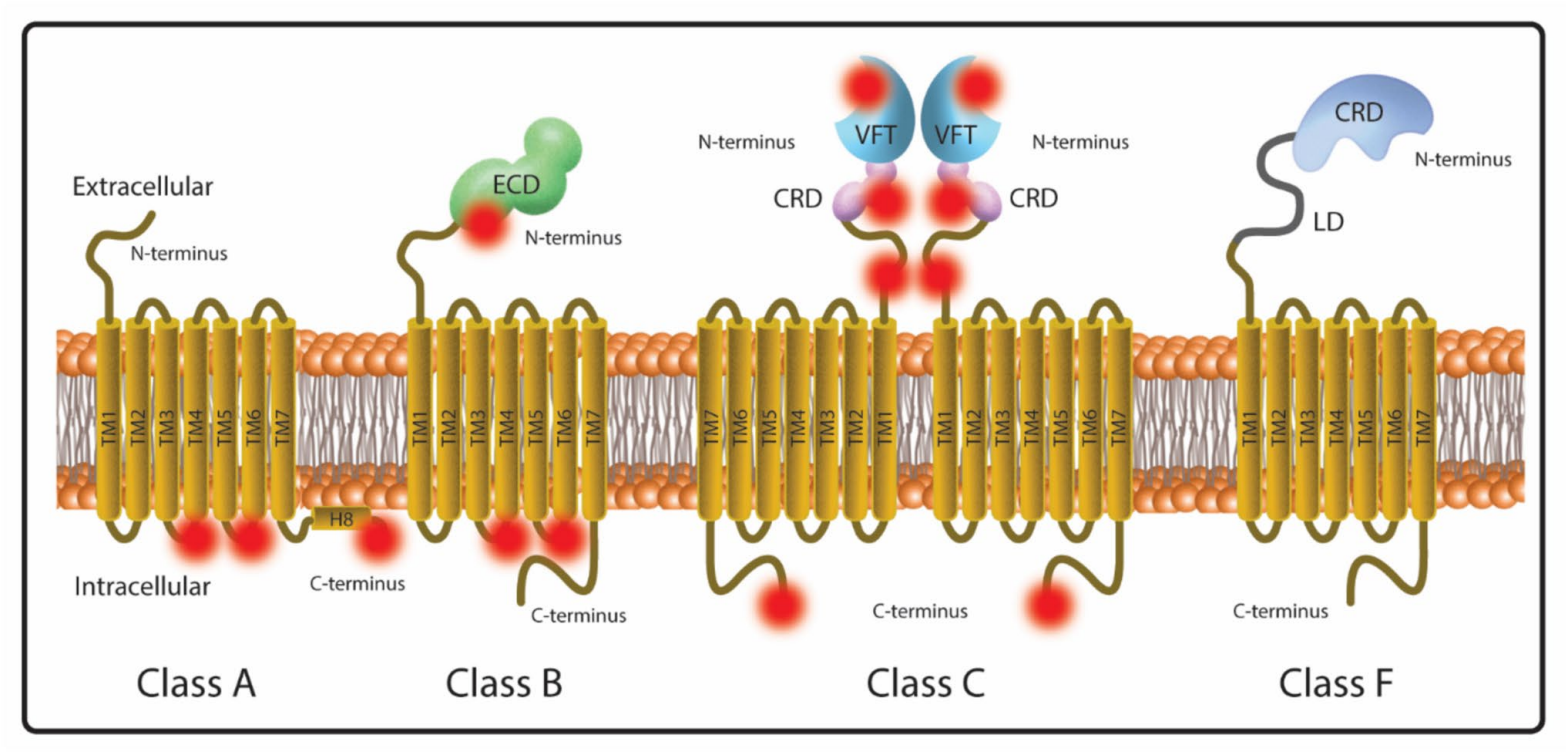
Structural diversity and typical labeling sites for different GPCR classes. The seven-transmembrane domain (7TMD) is characteristic of all GPCRs, but additional domains are present in different GPCR classes. Class A GPCRs contain only the 7TMD and a C-terminal helix 8 (H8), whereas other classes also feature additional extracellular domains (ECDs). Class B GPCRs possess a bulky ECD. The majority of Class C GPCRs contain a cysteine-rich domain (CRD) and a Venus flytrap (VFT) domain, also referred to as the ligand-binding domain (LBD). In Class F GPCRs, the ECD is composed of a linker domain (LD) and a CRD. Typical fluorescent labeling sites used in smFRET and smPIFE experiments are indicated as red dots. Class F GPCRs have not yet been labeled with fluorophores for smFRET or smPIFE studies, and therefore no labeling sites are shown

Single-molecule fluorescence techniques, such as smFRET and smPIFE, employ fluorescent dyes that are carefully placed at strategic positions within GPCR domains to monitor the conformational changes associated with allosteric signaling in these receptors. A variety of established approaches exist for site-specific labeling with fluorophores suitable for smFRET and smPIFE experiments, including cysteine-based labeling and the incorporation of unnatural amino acids, to name just a few (Agyemang et al. 2024; Krainer et al. 2019; Liauw et al. 2021; Quast & Margeat 2019; Tian et al. 2017). Since GPCRs are structurally diverse membrane proteins with distinct topologies, sequences, and domain architectures, tailored labeling strategies have been developed for each class to monitor the conformational changes underlying allosteric signaling. Commonly used labeling sites are illustrated in Fig. 3.

## Allosteric signaling phenomena of GPCRs studied by single-molecule fluorescence

Single-molecule techniques, including smFRET and smPIFE, have significantly advanced our understanding of the mechanisms underlying key aspects of allosteric signaling, such as ligand efficacy, biased signaling, and receptor modulation. In the following, we highlight major insights from smFRET and smPIFE studies that have deepened our knowledge of allosteric mechanisms in GPCRs. In each case, the receptor labeling sites and the Ballesteros–Weinstein numbers (superscripts) are indicated after each indicated TMD labeling positions. A summary of all published reports on GPCR signaling using smFRET and smPIFE is also provided in Table 1.

### Ligand efficacy

The extent to which a ligand activates a receptor defines its ligand efficacy (Kenakin 2002; Wacker et al. 2017). smFRET and smPIFE have been extensively used to study ligand efficacy effects in GPCRs. These studies, discussed below, have greatly enhanced our understanding of how different ligands influence receptor dynamics and initiate specific physiological responses in the cell.

#### Ligand efficacy in Class A GPCRs

**Fig. 4:**
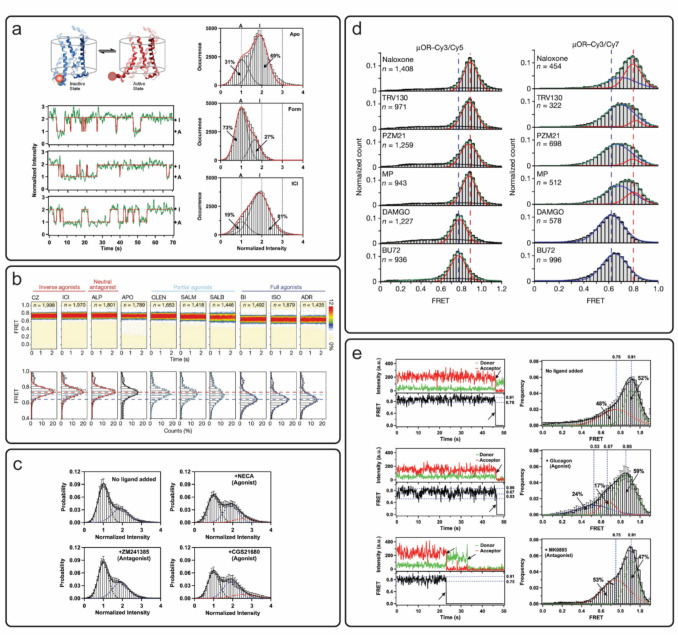
smFRET and smPIFE in the elucidation of ligand efficacy in GPCRs. (**a**) smPIFE trajectories and intensity histograms of β_2_AR in the absence or presence of different ligands. Images taken from Lamichhane et al. (2015). (**b**) smFRET histograms of β_2_AR. The motion of the TM6 of β_2_AR correlates with the efficacy of its ligands. Ligand efficacy also influences the formation and stabilization of β_2_AR-G_s_ complex. Images were taken from Gregorio et al. (2017). (**c**) smPIFE histograms of singly labelled A_2_AR in the unliganded form and in the presence of ligands of different efficacies. Images were taken from Wei et al. (2022). (**d**) smFRET histograms of the µ-opioid receptor (µOR) labelled with Cy3/Cy5 or Cy3/Cy7 fluorescent pairs and observed in the presence of different ligands with distinct efficacies, as well as the effect of ligand efficacy on the formation and stabilization of µOR-G_i_ complex. Images were taken from Zhao et al. (2024). (**e**) smFRET trajectories of the glucagon receptor (GCGR) in the presence of ligands with different efficacies. Image was extracted from Liu et al. (2023)

Early insights into the potential of smFRET and smPIFE for studying ligand efficacy came from Millar and colleagues (Lamichhane et al. 2015), who used smPIFE of Cy3 to investigate ligand-induced conformational dynamics of the β−2 adrenergic receptor (β_2_AR), a model Class A GPCR. They purified a minimal-cysteine β_2_AR construct from Sf9 insect cell membrane using n-Dodecyl-β-D-Maltoside (DDM) and cholesteryl hemisuccinate (CHS) and reconstituted it into lipid-bilayer nanodiscs encapsulated by membrane scaffold proteins (MSPs). The receptor was site-specifically labelled with Cy3 at position 265 (C265^6.27^) (the amino acids are numbered using the Ballesteros-Weinstein system (Ballesteros & Weinstein 1995) (see Fig. 3 for labeling locations), immobilized on quartz slide, and receptor dynamics was monitored with TIRF microscopy in the presence or absence of ligands of varying efficacies (Fig. 4a). Their findings revealed frequent transitions between two dominant states, a high-fluorescence intensity (inactive state) and a low intensity (active state), each with distinct substates. In the unliganded (apo) state, β_2_AR showed basal activity, occupying both active (~ 31%) and inactive (~ 69%) states. With the full agonist, formoterol, the inactive state was largely depopulated in favor of the active state (~ 73%), with increased activation frequency. In contrast, binding of the inverse agonist ICI-118551 shifted the equilibrium toward the inactive state (~ 81%), with reduced active state occupancy (~ 19%) and more frequent deactivation transitions. This study provided two key insights: (i) agonists redistribute existing conformational states rather than creating new ones, and (ii) even with full agonist binding, a mix of active and inactive states remains, consistent with prior ensemble studies using NMR (Liu et al. 2012; Nygaard et al. 2013).

Gregorio et al. (2017) conducted the first smFRET study to examine the role of ligand efficacy in β_2_AR activation. Using a minimal cysteine variant of β_2_AR solubilized using DDM, labeled with Cy3B and Cy5 at positions 266 in the TM6 (L266C^6.28^) and 148 in the TM4 (N148C^4.40^) (see Fig. 3 for labeling locations), and immobilized via an M1 Fab fragment, they investigated the effects of nine ligands with various efficacies via TIRF microscopy (Fig. 4b). With a neutral antagonist (alprenolol) and inverse agonists (carazolol, ICI-118,151), the receptor displayed high, stable FRET efficiencies (~ 0.74), similar to the unliganded state, indicating an inter-dye distance of ~ 4.2 nm. Partial agonists (clenbuterol, salmeterol, salbutamol) reduced FRET slightly (~ 0.71), while full agonists (adrenaline, isoproterenol, BI-167107) further lowered FRET (~ 0.64), suggesting an efficacy-dependent outward shift of TM6. The study also examined G protein binding effects. With both agonists and the G_s_ heterotrimer, a low-FRET state (~ 0.4 FRET, ~ 5.5 nm inter-dye distance) emerged, indicating an additional outward TM6 shift. The population and duration of this low-FRET state were efficacy-dependent, with full agonists enhancing β_2_AR-G_s_ complex stability more effectively than partial agonists. Decoupling of the β_2_AR-G_s_ complex by introducing GTP and GDP to the reaction medium revealed faster transitions from active (low-FRET) to inactive (high-FRET) states in the presence of full agonists. These results indicate that receptor conformational rearrangement plays a major role in mediating ligand efficacy effects, consistent with previous studies (Lamichhane et al. 2015; Olofsson et al. 2014). This ligand-dependent conformational rearrangement dictates the coupling of G_s_, with ligand efficacy directly influencing the rate of nucleotide exchange and receptor-G_s_ stability.

Recently, Casiraghi et al. (2025) identified three distinct β_2_AR states, inactive, intermediate, and active, using DY549PI/Alexa 647 fluorophores for improved signal quality. They solubilized a β_2_AR construct using a DDM/CHS mixture and performed a detergent exchange into a lauryl maltose neopentyl glycol (LMNG)/CHS mixture for smFRET measurements. In the apo state, the β_2_AR mainly occupied two high-FRET inactive states (~ 0.9 and 0.7 FRET) with occasional transitions to a low-FRET active state (~ 0.2 FRET). The addition of the full agonist, BI-167107, increased the intermediate-state population (~ 0.6 FRET), while the super agonist, LM189, further shifted the equilibrium toward the active state. This result further supports the dependence of receptor activation on the efficacy of interacting ligands, consistent with the findings of Blanchard and colleagues (Gregorio et al. 2017).

Beyond β_2_AR, another Class A GPCR, A_2_AR, has become a prominent model for single-molecule fluorescence studies. Fernandes et al. (2021) used smFRET to investigate ligand-induced conformational dynamics in detergent-solubilized A_2_AR as it diffused through a confocal volume. They labeled a double-cysteine variant of A_2_AR solubilized using LMNG and CHS, with Alexa Fluor 488 and Alexa Fluor 647, and monitored its dynamics with various ligands. In the unliganded (apo) form, A_2_AR showed a broad FRET distribution centered at ~ 30%, indicating two main FRET states: a low-FRET (active) state and a high-FRET (inactive) state. In the presence of an inverse agonist (ZM241385), the high-FRET state population increased by ~ 50%. With a partial agonist (LUF5834), the FRET distribution narrowed to an intermediate state at ~ 38%. The addition of a full agonist (NECA) shifted the distribution further toward a low-FRET active state, corresponding to a ~ 1.0 nm shift of TM6 relative to TM4, consistent with efficacy-dependent displacement of TM6. In addition to providing insights on the efficacy-dependent conformational rearrangement of A_2_AR similar to β_2_AR (Gregorio et al. 2017; Lamichhane et al. 2015), these results also showed that partial agonists are able to shift A_2_AR conformation to a highly dynamic intermediate state.

Wei et al. (2022) corroborated these findings by studying a A_2_AR-A289C variant solubilized in DDM/CHS and reconstituted into lipid nanodiscs that was surface immobilized for smPIFE measurements. They labeled A_2_AR at residue 289 (A289C^7.54^) with Cy3 (see Fig. 3 for labeling locations) and observed ligand-induced dynamics with different efficacies. In the unliganded and antagonist-bound states, A_2_AR exhibited two main fluorescence states (state 1 and state 2 with 62% and 38% intensity, respectively) (Fig. 4c). Two agonists, NECA and CGS21680, reduced the population of state 1 and induced a third fluorescence emission state (state 3). In the presence of NECA, state 1 and state 3 populations were 49% and 12%, respectively. CGS21680 produced a similar but more pronounced shift (state 1: 46%, state 3: 15%), indicating greater ligand efficacy. The population of state 2 remained unaffected by either agonist. The authors proposed that states 1, 2, and 3 represent inactive, intermediate, and active receptor conformations, with transitions occurring sequentially. The identification of a ligand-induced intermediate state for A_2_AR indicates the presence of multistate conformations that have been also reported for the β_2_AR (Casiraghi et al. 2025).

Maslov et al. (2023) further supported these observations, using smFRET to monitor ligand-induced changes between TM6 and H8 in A_2_AR reconstituted into nanodiscs. Using Alexa 488 and Atto 643 labels at residues 225 (L225C^6.27^) and 310 (Q310C^8.65^) (see Fig. 3 for labeling locations), they observed that the inverse agonist, ZM241385, maintained a FRET distribution similar to that of the apo state, while partial and full agonists shifted the FRET efficiency, favoring the active state. The authors identified three states, inactive, intermediate, and active, undergoing fast (sub-millisecond) transitions. Agonists populated the active state proportionally to their efficacy, while 10–20% of receptors remained in the inactive state. Together, these studies illustrate the efficacy-dependent conformational shifts in A_2_AR, with multiple studies reinforcing the presence of distinct inactive, intermediate, and active states, modulated by ligand binding.

Zhao et al. (2024) built on smFRET insights from β_2_AR and A_2_AR to investigate the efficacy of six ligands on the µ-opioid receptor (µOR), another Class A GPCR. A minimal-cysteine µOR construct with mutations R182C (R182C^4.38^)/R273C (R273C^6.26^) or T180C (T180C^34.56^)/R276C (R276C^6.29^) was labeled with Cy3/Cy5 or Cy3/Cy7 donor/acceptor fluorescent pairs (see Fig. 3 for labeling locations). This was solubilized in DDM–CHS that was exchanged to LMNG during affinity purification, immobilized on quartz, and observed via TIRF microscopy. While Cy3/Cy5-labeled µOR showed broad FRET distributions (Fig. 4d), it still revealed ligand-dependent shifts in receptor conformation. Increasing ligand efficacy, from the antagonist naloxone (~ 0.89 FRET) to the partial and G-protein biased agonists TRV130, MP, and PZM21, shifted the receptor equilibrium toward an active state. High-efficacy ligands, DAMGO and BU72, fully activated the receptor, reaching a low-FRET peak (~ 0.77 FRET). With Cy3/Cy7 labeling, µOR conformational states were better resolved: naloxone favored a high-FRET (~ 0.8) inactive state, while low-efficacy agonists partially populated a low-FRET active-like state (~ 0.7) proportional to their efficacy, following the order TRV130 > PZM21 > MP. High-efficacy agonists DAMGO and BU72 fully shifted µOR to the low-FRET state, indicating more efficient receptor activation. The authors suggested that the ~ 0.77 FRET state represents a time-averaged equilibrium of active and inactive µOR conformations. In addition to providing important information on the role of fluorescent pairs on smFRET output, these results showed that, similar to A_2_AR and β_2_AR, ligand efficacy critically modulates the conformational dynamics of µOR, indicating some conformational homogeneity among Class A GPCRs.

#### Ligand efficacy in Class B GPCRs

smFRET has also been used to study ligand-induced dynamics in Class B GPCRs. Liu et al. (2023) investigated the ECD dynamics of the glucagon receptor (GCGR), a Class B GPCR involved in blood glucose homeostasis. A GCGR construct with mutations L49C, C171A, and C401A was solubilized from human HEK 293 T cells using DDM-CHS and labeled at L49C (ECD) and C287 (C287^4.55^) (TMD) with Alexa 555/Alexa 647 donor/acceptor pairs (see Fig. 3 for labeling locations). In the unliganded (apo) state, GCGR exhibited transitions between two FRET states (~ 0.91 and 0.75 FRET), with calculated inter-dye distances of ~ 3.1 nm and ~ 4.3 nm, respectively, and similar behavior was observed with the antagonist MK0893 (Fig. 4e). In contrast, with the agonist glucagon, three distinct FRET states appeared at ~ 0.86, ~ 0.67, and ~ 0.53 FRET. The lower FRET states at ~ 0.67 and ~ 0.53, corresponding to inter-dye distances of ~ 4.4 nm and ~ 5.0 nm, respectively, were proposed to represent multiple open ECD conformations, with the lowest FRET state indicating a fully open ECD that allows glucagon to access the orthosteric pocket. This study provided two key insights: (i) The ECD of the unliganded receptor is dynamic and predominantly maintains a closed conformation and (ii) agonists induce a higher dynamic behavior on GCGR and the opening of ECD relative to the TMD.

#### Ligand efficacy in Class C GPCRs

A few studies have explored Class C GPCRs. The first smFRET experiment on a GPCR, conducted by Olofsson et al. (2014), examined the dynamics and activation of the isolated extracellular LBD of the metabotropic glutamate receptor 2 (mGluR2), a Class C GPCR involved in neurotransmission and synaptic regulation. They used SNAP-tagged mGluR2 LBD dimers produced in human HEK 293 cells and labeled with benzylguanine-Cy3b (donor) or benzylguanine-d2 (acceptor), to monitor conformational rearrangements between the two LBD protomers during transitions between the inactive/resting (R) and active (A) states in response to various ligands (see Fig. 3 for labeling locations). In the unliganded state, the LBD dimers mainly populated a medium-FRET state (~ 0.51), with smaller populations in high-FRET (~ 0.85) and low-FRET (~ 0.25) states. Ligand binding shifted the R ↔ A equilibrium in an efficacy-dependent manner: Full agonist glutamate decreased medium-FRET efficiency to ~ 0.42 and increased transitions to the low-FRET state, shifting the receptor to the A state. Antagonist LY341495 reversed these effects, shifting the receptor back to the R state. Partial agonists LCCG-1 and DGC-IV showed intermediate effects. These findings provided interesting insights about the ligand-induced dynamics of the LBD of mGluR2: (i) agonists influence the R ↔ A transition rate without fully stabilizing individual states; (ii) agonist efficacy correlates with their ability to shift the R ↔ A equilibrium towards the active state; and (iii) the free-energy barrier between the resting and active states is relatively low.

Vafabakhsh et al. (2015) extended this work to full-length mGluR2 and mGluR3 produced in HEK 293 T cells, solubilized with DDM and exchanged into octylphenoxypolyethoxyethanol detergent (IGEPAL) for TIRF microscopy. The receptors were labeled with the FRET pair DY-547 and Alexa-647 using amino-terminal SNAP or CLIP-tagged proteins to probe for ligand-induced structural rearrangements in the LBDs of the dimeric receptors (see Fig. 3 for labeling locations). For the unliganded mGluR2, they observed a high-FRET state (~ 0.45), which shifted to a low-FRET state (~ 0.2) in the presence of the agonist glutamate, with rapid transitions between high-, low-, and medium-FRET states at intermediate concentrations. The full agonist, LY379268, favored the low-FRET state more strongly than the partial agonist, DGC-IV, reflecting efficacy-dependent stabilization. The antagonist, LY341495, reversed agonist effects, restoring the high-FRET state. Unlike Olofsson's findings in isolated LBDs, Vafabakhsh et al. (2015) observed that agonists binding to full-length mGluR2 stabilized the active state proportionally to their efficacy. Similar results were seen in mGluR3, although its active state was more stable. These studies collectively highlight that ligand efficacy in Class C GPCRs influences state transitions and stabilization, with full-length receptors showing greater stabilization of active states in response to agonist.

Habrian et al. (2019) studied the ligand-induced conformational dynamics at the LBD of mGluR7 and mGluR2 using smFRET. They used receptor variants carrying an N-terminal SNAP tag produced in HEK 293 T cells, that were labeled with Alexa 647 (acceptor) and DY-547 (donor) fluorescent pairs (see Fig. 3 for labeling locations) and solubilized using IGEPAL. Similar to the findings of Vafabakhsh et al. (2015), mGluR2 settles at a high-FRET level of ~ 0.45 (resting state) at 0 μM glutamate, which transitions to a low-FRET level of ~ 0.2 (active state) at 1 mM glutamate. However, no detectable FRET change was observed in mGluR7 at 1 mM glutamate, while only rare, short-lived transitions to the low-FRET state were observed at 10 mM glutamate. At saturating glutamate concentrations of > 100 mM, only 10% of the receptor transitioned to the active state, suggesting that glutamate is a low efficacy agonist of mGluR7, consistent with previous reports (Okamoto et al. 1994). LSP4-2022, a synthetic agonist, however, showed higher potency at activating mGluR7, inducing frequent transitions to the low-FRET state with only 20 μM LSP4-2022, and ~ 65% activation with 3 mM of the agonist. The authors further investigated the activation mechanism of a mGluR7/mGluR2 heterodimer, building on the heterodimerization properties of mGluR7 (Doumazane et al. 2011; Shigemoto et al. 1997). They co-expressed SNAP-tagged mGluR7 with CLIP-tagged mGluR2 and labeled the heterodimer with BG-Alexa 647 (acceptor) and BC-DY-547 (donor) (see Fig. 3 for labeling locations), to monitor conformational changes at the LBD. Their analysis showed that ~ 50% of the mGluR7/2 heterodimer occupied the active state using only 10 μM glutamate, which increased to ~ 85% using 10 mM glutamate. These findings indicate that heterodimeric interaction between mGluR7 and mGluR2 more strongly favors the activated state than homodimeric mGluR7 interaction, further expanding our understanding of the complexity of Class C GPCR allosteric signaling.

Asher and his team extended these findings on mGluRs by elucidating the ligand-induced conformational dynamics of mGluR2 in living cells, marking an advancement in smFRET imaging and the elucidation of receptor allosteric signaling (Asher et al. 2021). They used a variant of mGluR2 bearing an N-terminal SNAPfast tag, expressed in CHO cells, and labelled with lumidyne 555p (donor) and lumidyne 655 (acceptor) fluorescent pairs (see Fig. 3 for labeling locations) to monitor glutamate-induced dynamics of the LBD of mGluR2 within the plasma membrane using smFRET. Their results showed that, in the absence of ligand (apo), the receptor predominantly populated a high-FRET state at ~ 0.46 FRET. In the presence of the full agonist, glutamate, receptors transited to a low-FRET state at ~ 0.29 FRET, consistent with previous reports (Doumazane et al. 2011; Olofsson et al. 2014; Vafabakhsh et al. 2015). Population of receptors in this low-FRET state increased with increasing glutamate concentration, and direct but rare transitions were observed between the low-FRET and high-FRET states in the presence of glutamate, confirming agonist-induced conformational changes within the LBD. In addition to establishing the potential of smFRET for elucidating receptor dynamics within living cells, these results further illuminate the ligand-induced conformational processes in Class C GPCRs.

Liauw et al. (2021) used smFRET to study the propagation of conformational changes during the activation of mGluR2 (Liauw et al. 2021). They used a mGluR2 construct containing an unnatural amino acid (UAA) at position 548 in the CRD and a C-terminal FLAG tag, expressed in HEK 293 T cells. The receptor was labeled in cells using Cy3 (donor) and Cy5 (acceptor) fluorescent dyes (see Fig. 3 for labeling locations), solubilized with detergent (DDM/CHS) and immobilized for smFRET analysis using an anti-FLAG tag antibody. Their results showed that, in the absence of an agonist or in the presence of LY34149 antagonist, the CRD domain of the receptor displayed rapid transitions among four major FRET states, with peaks at FRET efficiencies of 0.31, 0.51, 0.71, and 0.89 – markedly different from the low conformational dynamics observed in the VFT domain of the same receptor (Vafabakhsh et al. 2015). In the presence of glutamate, occupancy of the four FRET states shifted toward the higher FRET states in a concentration-dependent manner. Notably, the receptor remained highly dynamic with constant transitions occurred in a sequential manner between adjacent FRET peaks. In the presence of other mGluR2 agonists, such as DGV-IV and LY37, the CRD retained population across the four FRET states. However, LY37 showed ~ 21% higher occupancy of the high FRET state at 0.89 compared to glutamate, while in the presence of DGC-IV the occupancy of this state was ~ 23% lower, consistent with their ligand efficacies. The FRET peak at 0.31 was attributed to the inactive state conformation of the CRD, while the high FRET peak at 0.89 was assigned to the active state conformation of the CRD. These results suggest that the CRD of mGluR2 is highly dynamic and undergoes conformations changes distinct from those of the VFT domain, indicating that different domains of the same receptor can exhibit separate conformational processes, highlighting the conformational heterogeneity of Class C GPCRs.

Lecat-Guillet et al. (2023) further investigated the ligand-induced conformational landscape of the mGluR2 receptor. They incorporated the UAA PrF at positions 248 and 358 in the LBD of mGluR2 expressed in HEK 293 T cells, to monitor ligand-induced closure of the LBD domains. The receptor was labeled in crude membranes with Alexa Fluor 488-picolyl-azide (donor) and Alexa Fluor 647-picolyl-azide (acceptor) (see Fig. 3 for labeling locations) followed by solubilization using LMNG/CHS detergent mix and subsequent dilution of the protein into a buffer supplemented with glycol-diosgenin (GDN) for smFRET analysis. In the absence of ligand or presence of LY341495 antagonist, this mGluR2 variant populated a low-FRET state centered at ~ 0.23 FRET (open conformation). The partial agonist, DCG-IV, shifted ~ 50% of the receptors to a high-FRET state centered at ~ 0.56 FRET (closed LBD conformation), while the full agonist, glutamate, moved ~ 80% of the receptors to this high-FRET. Another construct of the receptor, bearing a PrF probe only at position 358 at the upper lobe of the LBD was further analyzed to monitor the reorientation of the upper lobes of the LBD. ~ 95% of this construct populated a high-FRET state centered at ~ 0.6 FRET (open conformation of the LBD upper lobe) in the absence of ligand and presence of LY341495 antagonist. In the presence of DCG-IV, about 35% of the receptors populated a low-FRET ~ 0.3 state (active, closed conformation of the LBD upper lobe), while glutamate increased active state population to ~ 60%, similar to reorientation events observed at the lower lobe of the LBD. These results suggest that ligands lead to different conformations of the active and resting state of the LBD in mGluR2, with stabilization of the active conformation dependent on ligand efficacy.

In a more recent study, Latorraca and his colleagues investigated the activation pathway triggered by glutamate in mGluR2 (Latorraca et al. 2025). They co-transfected a mGluR2 variant bearing UAAs—with amber stop codons at sites S463 and Q359 to monitor LBD closure or at a single site A248 to monitor LBD twisting—with an unaltered mGluR2 construct that carries an N-terminal HA tag. These constructs were expressed in HEK 293 T cells, labelled with pyrimidyl-tetrazine-AF555 and pyrimidyl-tetrazine-AF647 fluorescent pairs (see Fig. 3 for labeling locations) before the receptor was extracted from the membrane using MNG/GDN/CHS detergent mixture for TIRF-smFRET analysis (Latorraca et al. 2025). LBD closure analysis showed a stable, narrow, low-FRET distribution, centered at ~ 0.38 FRET (open LBD conformation) at 0 mM glutamate. With 10 mM glutamate, the population was shifted to a high-FRET state centered at ~ 0.63 FRET (closed LBD conformation), while an intermediate glutamate concentration of 10 μM broadened and centered the distribution at ~ 0.5 FRET, caused by rapid interconversion between the open and closed LBD conformations at equal rates. smFRET analysis of LBD twisting showed a broad low-FRET (relaxed LBD) distribution at ~ 0.18 FRET with 0 mM glutamate, which transited to an unstable high-FRET (twisted LBD) state at ~ 0.44 FRET with 10 mM glutamate. At an intermediate glutamate concentration of 10 μM, transitions between the open and closed conformation of the LBD occurred at equal rates. approximately 37% of receptors populated the twisted LBD state with transitions between the relaxed and twisted LBD occurring very slowly. Even at saturating glutamate concentration (10 mM), which induces full closure of the LBDs, domain twisting of the LBDs persisted. These findings indicate that glutamate-induced LBD closure and domain reorientation are only loosely coupled processes. Furthermore, full activation of mGluR2 requires agonism of both LBDs and is minimal when only one of the LBD dimers is activated, consistent with previous reports (Kunishima et al. 2000; Lecat-Guillet et al. 2023).

Latorraca et al. (2025) further investigated how rearrangements at the LBD in mGluR2 couples to conformational changes at the CRD of the receptor. For this, they used a variant of mGluR2 that carries an amber stop codon at A548 approximately in the middle of the CRD linker (see Fig. 3 for labeling locations) and expressed/labelled it as described above to monitor conformational rearrangements and intersubunit distances between the CRD of each mGluR2 homodimer. Their smFRET analysis of this variant showed that in the absence of glutamate, the CRD populated a low-FRET state at ~ 0.28 FRET (resting CRD conformation), while the presence of glutamate led to population of a medium-FRET state centered at ~ 0.48 FRET (proximal CRD conformation) with an occupancy that increases with increasing glutamate concentration. They observed that at all concentrations of glutamate, the LBD adopted a high-FRET active conformation more often than the CRD adopted the medium-FRET proximal conformation, suggesting that the twisting of the CRD lags behind the reorientation of the LBD. By using a high-affinity agonist, LY379268, they observed a higher population of receptors adopting the CRD proximal conformation than when glutamate was used. They concluded that, although the LBD clamshell is closed and the LBD lobes are twisted, the CRD of mGluR2 can remain in a resting (inactive-like) conformation, even at saturating glutamate concentration, and full activation is only possible in the presence of G proteins.

### Functional selectivity and biased signaling

GPCRs display varying degrees of selectivity for different G protein isoforms and can exhibit signaling bias towards distinct transducers, such as arrestins and kinases, depending on the ligand bound to the receptor. Biased ligands can enhance binding of specific transducers, selectively activating one signaling pathway over others. Investigating functional selectivity or biased signaling involves examining receptor activation mechanisms in the presence of different ligands and various transducers or their isoforms. Such studies have been conducted using various in vivo approaches and in vitro ensemble techniques, including DEER (Casiraghi et al. 2025; Manglik & Kobilka 2014; Wingler et al. 2019) and bioluminescence resonance energy transfer (BRET) (Avet et al. 2022; Eiger et al. 2022). smFRET and smPIFE have been used to study ligand-biased transducer recruitment, providing valuable insights into how biased signaling occurs at the molecular level.

**Fig. 5:**
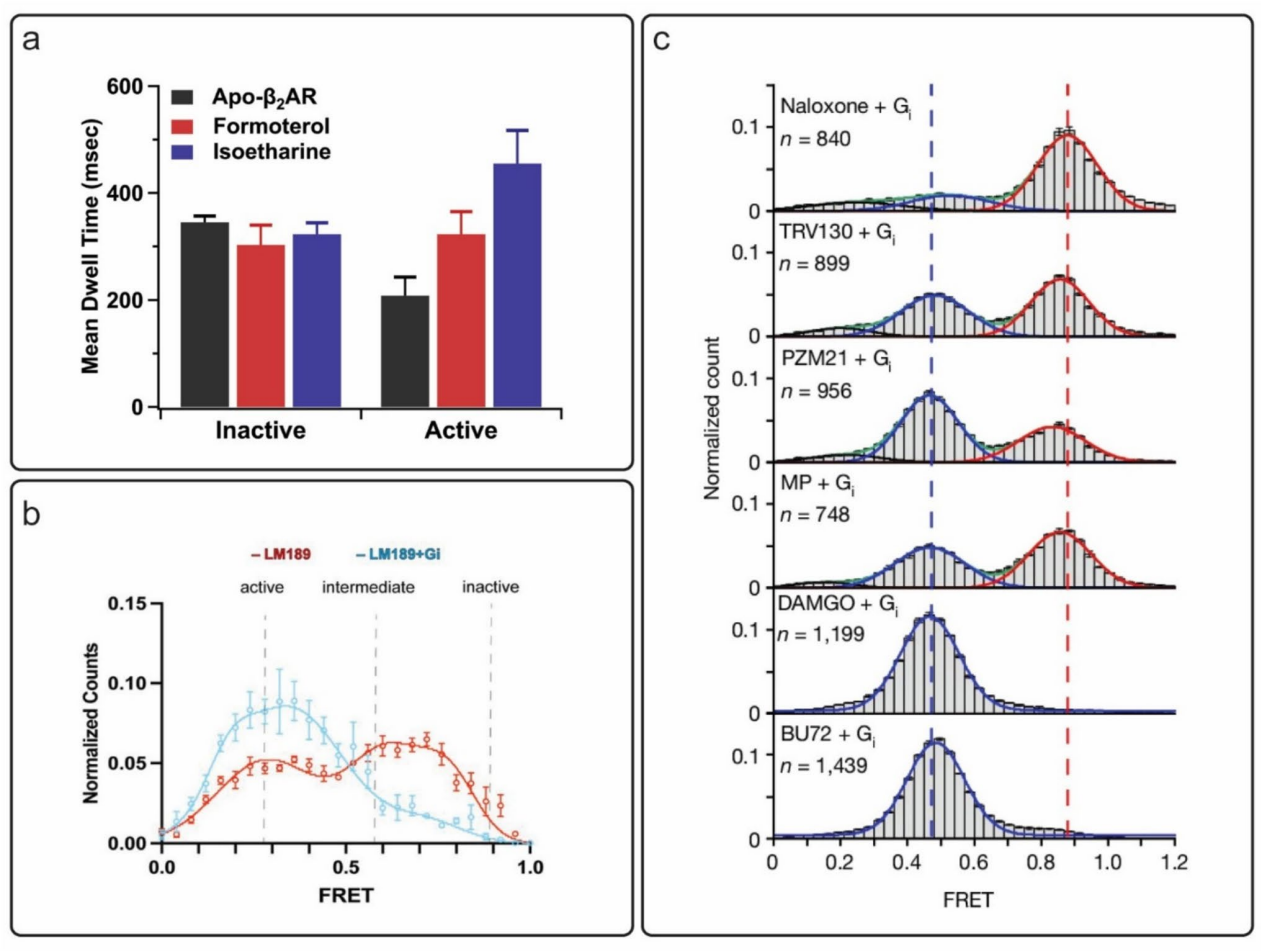
smFRET and smPIFE offer mechanistic insights into the biased signaling and functional selectivity of GPCRs. (**a**) Ligand bias influence mean dwell time of the active-like conformational state of β_2_AR. Images were taken from Lamichhane et al. (2020). (**b**) G_i_-biased agonism of LM189 in β_2_AR. Images were taken from Casiraghi et al. (2025). (**c**) smFRET distribution of µOR-G_i_ complex in the presence of different G protein-biased ligands. Images were taken from Zhao et al. (2024)

Lamichhane et al. (2020) conducted the first smPIFE study on biased signaling using the Class A GPCR, β_2_AR, as model receptor. They examined the effects of a balanced agonist, formoterol, and a β-arrestin-biased agonist, isoetharine. To track TM7 dynamics, they used a β_2_AR construct extracted from the cell membrane of Sf9 insect cell using DDM/CHS, reconstituted into MSP nanodiscs containing synthetic phospholipids, and labeled with Cy3 at C327 (C327^7.54^) (see Fig. 3 for labeling locations) via smPIFE, notably without including β-arrestin itself (Lamichhane et al. 2020). Building on prior findings that TM7 mediates signaling bias and β-arrestin binding (Che et al. 2018; Kang et al. 2015; Liu et al. 2012; McCorvy et al. 2018; Rahmeh et al. 2012), the authors identified two states: a high fluorescence intensity (inactive) state and a low fluorescence intensity (active) state. In the unliganded β_2_AR, the receptor predominantly occupied the inactive state, but both agonists shifted TM7 toward the active state. Analysis of transition kinetics and dwell times revealed that, while the inactive state’s dwell time was unaffected by either agonist, the active state dwell time increased with ligand efficacy (Fig. 5a). Isoetharine, the β-arrestin-biased agonist, notably extended the active state dwell time more than formoterol, suggesting a prolonged interaction window for β-arrestin engagement (Fig. 5a), potentially promoting a distinct signaling pathway. These findings reveal that ligands can induce signaling bias in GPCRs by simply prolonging the dwell time of the receptor in a particular state or modifying the kinetics of receptor conformational exchange through a series of highly complex mechanisms.

Recently, Casiraghi et al. (2025) conducted a study using smFRET to examine functional selectivity in β_2_AR. They labeled a minimal-cysteine β_2_AR variant, extracted from Expi293 cell membrane using DDM/CHS and detergent-exchanged into LMNG/CHS. The receptor was labeled on the intracellular sides of TM4 and TM6 with DY549P1 and Alexa 647 fluorophores (see Fig. 3 for labeling locations). They then used TIRF microscopy to monitor interactions of the receptor with two different G protein isoforms at the single-molecule level (Casiraghi et al. 2025). Their findings showed that the partial agonist, salmeterol, and the full agonist, BI167107, favored activation of the adenylyl cyclase pathway via stimulatory G protein (G_s_), while the agonist, LM189, showed a bias for the inhibitory G protein (G_i_) (Fig. 5b). When G_i_ was introduced to LM189-bound β_2_AR, TM6 shifted almost completely to a low-FRET state (Fig. 5b), corresponding to a ~ 4.6 nm distance between TM4 and TM6. Although smFRET data for LM189 with G_s_ was not provided, the authors suggested that LM189 stabilizes a G_i_-competent conformation distinct from that induced by G_s_ or other agonists, indicating that β_2_AR can selectively bind G_s_ or G_i_ depending on the ligand. This study suggested the mechanism behind the selective recruitment of different G protein isoforms by different ligands binding to the same receptor.

In addition to studying the efficacy of different ligands of the µ-opioid receptor (µOR) (Zhao et al. 2024) (see *Ligand efficacy in Class A GPCRs*), Zhao and his team further examined ligand efficacy in G protein binding to the Cy3/Cy5-labeled µOR receptor (see Fig. 3 for labeling locations). In this case, they monitored the influence of the antagonist, naloxone, the partial and G-protein-biased agonists TRV130, MP, and PZM21, and the high-efficacy ligands, DAMGO and BU72, on the µOR-G_i_ complex. Two distinct FRET states were reported: a high ~ 0.77 FRET state, which was apparent in the presence of antagonist and partial agonists, and a low-FRET ~ 0.5 state, representing full µOR activation (Fig. 5c). The population of this low-FRET state increased with ligand efficacy, following BU72/DAMGO > PZM21 > TRV130 > MP. Thid study suggested that TRV130, MP, and PZM21, DAMGO, and BU72 are G protein-biased agonists.

### Allosteric modulation

Allostery, the modulatory effect caused by the binding of small molecules, ions, and lipids, to sites on GPCRs other than the orthosteric site, is an important mechanism of GPCR signaling and pharmacological intervention. Allosteric modulation depends on the intrinsic properties of the modulators and the specific allosteric binding sites they occupy. This makes smFRET and smPIFE techniques versatile tools in unraveling the complex mechanisms involved in both allosteric and orthosteric interactions, as well as the impact on transducer coupling and downstream signaling.

**Fig. 6:**
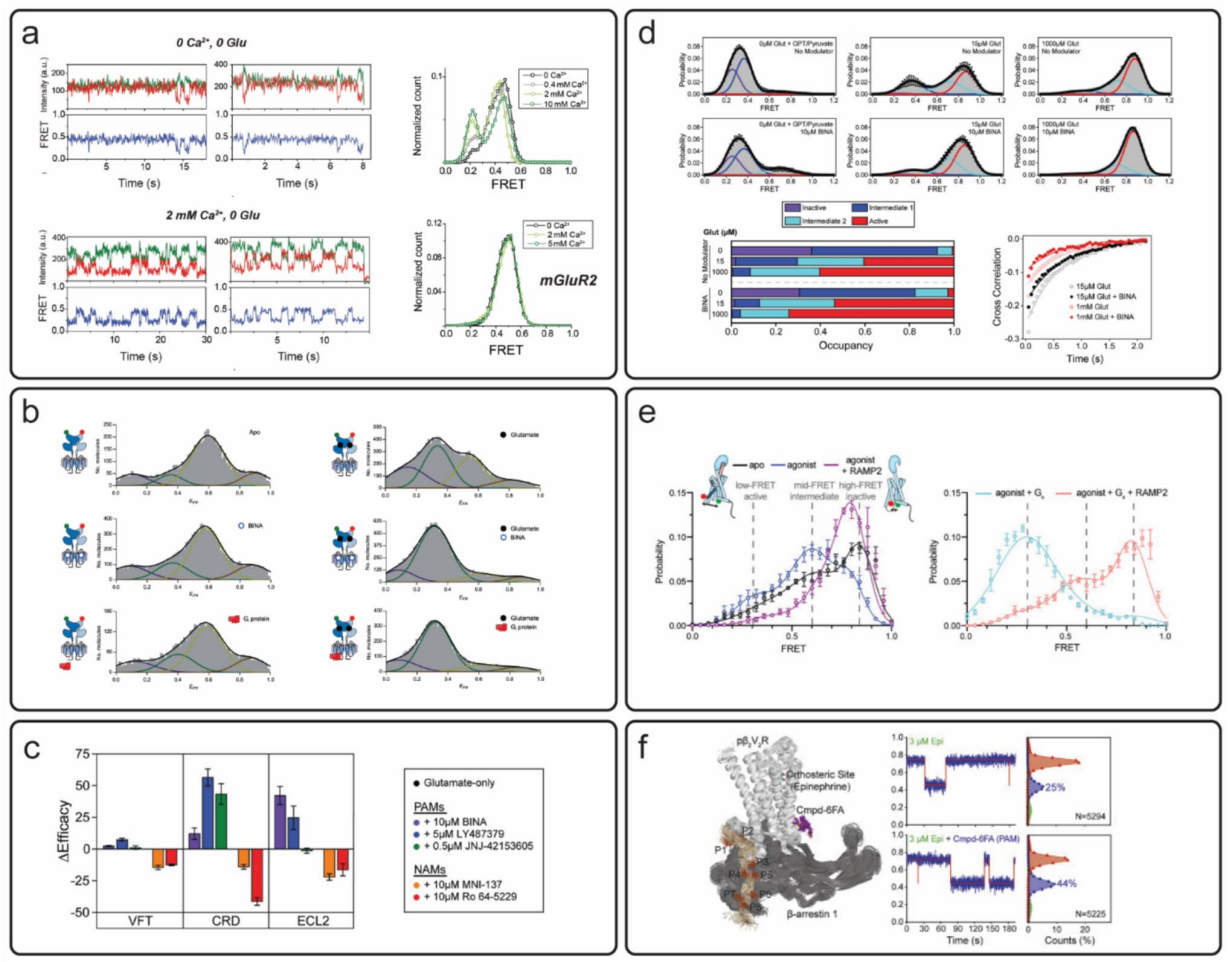
smFRET in deciphering the mechanism of allosteric modulation in GPCRs. (**a**) Influence of varying concentrations of Ca^2+^ on mGluR3, smFRET traces of mGluR3 response to Ca^2+^ in the absence of glutamate agonist, and irresponsiveness of mGluR2 to Ca^2+^. Images were taken from Vafabakhsh et al. (2015). (**b**) Representative histograms of doubly labelled mGluR2 molecules in the unliganded (apo) form, with BINA alone, with G_i_ protein alone, with glutamate agonist alone, with glutamate and BINA, as well as with glutamate and G_i_ protein. Images were taken from Cao et al. (2021). (**c**) Changes in glutamate efficacy in response to PAMs and NAMs on mGluR2 as measured by the different conformational sensor at the LBD, CRD, and 7TMD. Images were taken from Liauw et al. (2022). (**d**) Mean occupancy of four conformational states in the presence of the agonist-PAM, BINA compared to glutamate agonist obtained by monitoring conformation at the CRD. Images were taken from Liauw et al. (2022). (**e**) Negative allosteric modulation induced by RAMP2 on the glucagon receptor, GCGR. Images were taken from Kumar et al. (2023). (**f**) Positive allosteric modulation induced by Cmpd-6FA on β_2_AR. Images were taken from Asher et al. (2022)

The first investigation into allosteric modulation of GPCRs using smFRET was conducted by Vafabakhsh et al. (2015), who studied ion modulation of Class C GPCRs, mGluR3 and mGluR2, using TIRF microscopy. Building on earlier reports of Ca^2+^ sensitivity of mGluRs (Kubo et al. 1998), they created mGluR2 and mGluR3 variants with N-terminal CLIP and SNAP tags on the LBD, extracted them from HEK 293 T cell membranes using DDM, labeled them with DY-547 (donor) and Alexa 647 (acceptor) (see Fig. 3 for labeling locations), and immobilized them for smFRET-TIRF analysis in IGEPAL containing buffer. Varying the Ca^2+^ concentration influenced mGluR3, leading to a Ca^2+^-dependent increase in the low-FRET active state and transient occupancy of a medium-FRET intermediate state (Fig. 6a), while mGluR2 remained unaffected under similar conditions. Although Ca^2+^ was not required for full mGluR3 activation, it stabilizes the active state of the receptor, contributing to the receptor’s basal activity in the absence of ligands. This finding portrays the role of cellular ions in regulating basal activation and signaling of Class C GPCRs under physiological conditions. Of note, other GPCR families, particularly Class A GPCRs, are also modulated by monovalent and divalent ions such as sodium and magnesium, respectively. These ions influence ligand binding and receptor activation, necessitating careful control of assay conditions for spectroscopic studies aimed at exploring the conformational landscape of GPCRs (Burgmer et al. 1998; X. Hu et al. 2020; Johansson et al. 1992; Katritch et al. 2014; Rodriguez et al. 1992).

Tora et al. (2018) challenged these findings, using smFRET and time-resolved ensemble FRET approaches to show that mGluR3 is primarily potentiated by Cl^−^ rather than Ca^2+^. Their study demonstrated positive cooperativity between Cl^−^ and glutamate in the extracellular domain, forming a “Cl^−^ lock” that reinforces glutamate binding. Contrary to earlier reports suggesting Cl^−^ as a direct agonist (DiRaddo et al. 2015), Tora et al. found that Cl^−^ does not directly activate mGluR3 but instead enhances its response to ambient glutamate. This may indicate that the response of mGluR3 to Ca^2+^ reported in earlier studies of Vafabakhsh et al. (2015) may be due to Cl^−^ which is present in the CaCl_2_ they used as a source of Ca^2+^.

More recently, Cao et al. used smFRET to investigate the allosteric modulation of mGluR2 by the positive allosteric modulator, BINA, which binds to the transmembrane domain of the receptor (Cao et al. 2021). They used a SNAP-tagged mGluR2 construct labeled with Cy3b and d2 fluorophores at the LBD (see Fig. 3 for labeling locations), which was detergent-solubilized and monitored in a confocal microscope as it diffused through a confocal volume. In its unliganded (apo) form, mGluR2 exhibited a broad FRET distribution, with the majority populating a high-FRET (~ 0.6) inactive state (Fig. 6b). Saturating glutamate concentrations shifted the receptor to a low-FRET (~ 0.34) active state. BINA or G protein (G_i_) alone had minimal effect on receptor dynamics, indicating that BINA does not show any intrinsic agonist activity. However, in the presence of glutamate, BINA significantly depopulated the high-FRET state by ~ 85%, similar to the effect observed with G_i_ (Fig. 6b). The low-FRET active state stabilized by BINA or G_i_ fell on a static FRET line, unlike the high-FRET state, which showed sub-millisecond oscillations, suggesting BINA and G_i_ stabilize the LBD domain’s active state through allosteric modulation. Agonist titration experiments and smFRET analysis confirmed that BINA acts as a positive allosteric modulator, enhancing glutamate potency by stabilizing the active state of the 7TM domain, whose activation-linked reorientation appears to allosterically modulate the relative orientation of the LBD. This study provided the first insight into allosteric modulation of GPCR dynamics by small-molecule PAMs at the single-molecule level and revealed that PAMs can stabilize the active state of the LBDs to the same extend as heterotrimeric G proteins.

Liauw et al. (2022) expanded on Cao’s findings by using smFRET to examine the effects of three PAMs, BINA, LY487379, and JNJ-4215360, and two NAMs, MNI-137 and Ro 64–5229, on glutamate-bound mGluR2. They assessed how these modulators influenced conformational changes of SNAP-tagged mGluR2 solubilized in LMNG/CHS and labeled with Alexa Fluor 549, Alexa Fluor 647, Cy3, or Cy5 at the LBD, CRD, and TMD using SNAP-tags or unnatural amino acids (see Fig. 3 for labeling locations). The PAMs increased glutamate’s potency and efficacy in inducing conformational changes across all three domains, especially at the CRD and TMD, suggesting distinct modulatory mechanisms for each domain (Fig. 6c). In contrast, NAMs reduced glutamate efficacy, particularly at the CRD and TMD, aligning with previous reports that high NAM concentrations inhibit mGluR2 signaling (Hemstapat et al. 2007; Kolczewski et al. 1999). Further, Liauw et al. investigated whether BINA could activate mGluR2 independently of glutamate. Using smFRET on a C-terminal FLAG-tagged variant of mGluR2 labeled at the CRD, they found that BINA increased active-state populations in a concentration-dependent manner, surpassing glutamate’s effects (Fig. 6d). This finding indicates that BINA acts as an agonist-PAM, contradicting the conclusion of Cao et al. (2021), which suggests that the role of PAMs on GPCR allosteric signaling can go beyond simply allosteric modulation to allosteric activation similar to the effects observed in orthosteric agonists.

In addition to the findings of Liauw et al. (2022), Lecat-Guillet and colleagues further investigated the effect of allosteric modulators on the concerted conformational changes of full-length mGluR2 using smFRET (Lecat-Guillet et al. 2023). Using a variant of mGluR2 previously described (see *Ligand efficacy in Class C GPCRs*), the authors investigated the effect of the PAM, BINA, on the ligand-induced closure of the LBD of the receptor. They observed that active state population of the LBD increased from ~ 50% (with the partial agonist, DCG-IV alone) to ~ 75% when BINA was added. Interestingly, BINA did not exert any significant effect on glutamate-bound mGluR2. The authors further investigated a construct of the receptor fluorescently labelled at the upper lobe of the LBD (see *Ligand efficacy in Class C GPCRs* for description), to monitor the effect of BINA on the ligand-induced reorientation of the LBD upper lobe. They observed that the active state population of the LBD upper lobe increased from ~ 35% (with DCG-IV alone) to ~ 80% when BINA was added, while active state population in the presence of glutamate increased from ~ 55% to ~ 90% when BINA was added. These results suggest that while the full agonist glutamate can induce maximal LBD closure independently of an allosteric modulator, the full reorientation of the LBDs of mGluR2 is favored only when a positive allosteric modulator is bound to the TMDs, highlighting the complex mechanism of allosteric modulation of mGluRs.

Latorraca et al. (2025) further investigated the modulation of the glutamate-induced LBD twisting of mGluR2 by BINA using a mGluR2 variant expressed, solubilized, and labeled as previously described (see *Ligand efficacy in Class C GPCRs*). Their results showed that, although the presence of glutamate alone could not stabilize the twisted (active) conformation of the LBD of the receptor, introduction of BINA fully stabilized this active conformation, increasing population from ~ 55% (without BINA) to ~ 97% (with BINA) similar to the effect of introducing G protein, consistent with the findings of Cao et al. (2021). By monitoring FRET changes between fluorophores attached to the CRDs of the receptor (see *Ligand efficacy in Class C GPCRs*), the authors reported that, in the absence of BINA, the receptor adopts a conformation with closed LBD clamshells and twisted lower LBD lobes, while the CRDs remained in a resting (inactive) conformation. In the presence of BINA, the CRD populated an intermediate-FRET state centered at ~ 0.6, which was also observed in addition to a high-FRET (active) state at ~ 0.85 when G protein was introduced. These results suggest that the twisting of the LBDs and CRD reorientation are loosely allosterically coupled, and that binding of the PAM BINA to the TMD attenuates sampling of the active twisted LBD state and the CRD conformation, similar to G protein binding, indicating that PAMs stabilize mGluR2 in a conformation favorable for G protein coupling.

Another exploration into the allosteric modulation of receptors with bulky extracellular domains was conducted by Kumar et al. who examined the role of receptor activity-modifying protein-2 (RAMP2) on the glucagon receptor (GCGR), a Class B GPCR, using smFRET (Kumar et al. 2023). They labeled a minimal-cysteine GCGR variant extracted using LMNG/CHS with LD555 (donor) and LD655 (acceptor) at the cytoplasmic end of TM4 and the activation-sensitive TM6 (see Fig. 3 for labelling locations), followed by immobilization for TIRF microscopy. In the unliganded state, GCGR showed a broad FRET distribution with a major high-FRET peak (~ 0.83, inactive) and occasional transitions to a mid-FRET intermediate state (~ 0.63) (Fig. 6e). With a full agonist, the receptor shifted from high-FRET to populate mid- and low-FRET active states (~ 0.32 FRET). Adding RAMP2 to the agonist-bound receptor almost fully repopulated the high-FRET inactive state, effectively deactivating the receptor. Similarly, RAMP2 reduced the low-FRET active state in the G_s_-coupled receptor, indicating that RAMP2 binding deactivates the agonist- and G_s_-bound GCGR, reducing the likelihood of productive GCGR-G_s_ formation. This study revealed that RAMP2 exerts a negative allosteric effect on GCGR signaling by disordering the ECD of the receptor resulting in the inhibition of the active and intermediate states, signifying a unique dynamic allostery mechanism by which the protein acts as a NAM to deactivate the receptor.

Allosteric modulation of β-arrestin activation in a Class A GPCR was investigated by Asher et al. (2022) using smFRET. They studied the role of a positive allosteric modulator, Compound-6FA (Cmpd-6FA), in facilitating β-arrestin activation by examining a β_2_V_2_R chimera in LMNG. In this construct, the C tail of β_2_AR was replaced by that of the V2 vasopressin receptor to increase its affinity for β-arrestin-1 (Asher et al. 2022). By immobilizing the β-arrestin-1 E176C/K397C variant labeled with LD555-MAL (donor) and LD655-MAL (acceptor) and incubating it with 1 µM β_2_V_2_R, they found that Cmpd-6FA binding to an allosteric site near intracellular loop 2 (ICL2) of the receptor significantly enhanced epinephrine's ability to activate β-arrestin (Fig. 6f). Cmpd-6FA nearly doubled the population of the active state and extended the active-state dwell time compared to epinephrine alone, indicating that PAMs can enhance β-arrestin recruitment and activation by β_2_AR. This study revealed the mechanism by which PAMs modulate the conformation of GPCRs towards transducer recruitment and full receptor activation that affords downstream signaling.

## Conclusions and Outlook

The intricate allosteric processes in GPCRs govern a majority of the physiological activities within cells. Over the years, single-molecule fluorescence techniques, particularly smFRET and smPIFE, have significantly advanced our understanding of these mechanisms by providing detailed molecular-level insights. While these approaches have shed light on the mechanistic dynamics of several GPCRs, critical gaps remain in our understanding of the allosteric signaling phenomena in these physiologically and pharmacologically crucial proteins.

Although smFRET and smPIFE have provided substantial insights into the response of Class A, B, and C receptors to ligands of various efficacies at the single-molecule level, their potential has yet to be fully leveraged for studying Class F GPCRs. This receptor class includes ten Frizzled receptors (FZD_1-10_) and the Smoothened receptor (SMO) (Schulte & Kozielewicz 2020), which play critical roles in regulating cell proliferation, polarity, and differentiation. Class F GPCRs are essential for the Wingless-related integration (Wnt) and Hedgehog (Hh) signaling pathways (Briscoe & Thérond 2013; Logan & Nusse 2004) and are implicated in various diseases, including birth defects, skin disorders, and cancers (Arensdorf et al. 2016; Xie et al. 1998). Extending smFRET and smPIFE techniques to unravel the ligand-induced dynamics of Class F receptors could provide transformative insights into their mechanisms, paving the way for more effective therapeutic strategies (review on Class F receptors including a discussion of receptor dynamics: (Schulte 2024)).

In addition, the smFRET and smPIFE data on the mechanisms underlying biased signaling remain scarce. For instance, the selective recruitment of G_s_ (stimulation of adenylyl cyclase) over G_i_ (inhibition of adenylyl cyclase) or G_q/11_ (stimulation of phospholipase C), and vice versa, has yet to be explored at the single-molecule level. Similarly, smFRET and smPIFE have not been directly employed to study the biased recruitment of G proteins over arrestins, or the reverse. Furthermore, the allosteric mechanisms driving the phosphorylation of the GPCR C-terminal tail by GRKs, which triggers G protein dissociation (receptor desensitization) and arrestin recruitment, remain unexplored using these techniques. Investigating these processes at the single-molecule level through the advanced capabilities of smFRET and smPIFE holds great potential for advancing our understanding and aiding the development of highly active and selective therapeutic agents.

Furthermore, allosteric modulation of GPCRs has opened new avenues for developing targeted GPCR drugs (Hauser et al. 2017; Leach et al. 2007; Wootten et al. 2013). Despite its significance, allosteric modulation has been minimally explored at the single-molecule level for Class A and B GPCRs, and no studies have yet investigated Class F GPCRs in this context. This is surprising given ensemble experiments (Gater et al. 2014; Gimpl et al. 1997; Hall et al. 2019; Levitt et al. 2009; Pan 2001) and structural data (Bueno et al. 2020; Liu et al. 2020; Oswald et al. 2016; Song et al. 2017) that highlight the modulation of these receptors by cholesterol. Leveraging the capabilities of smFRET and smPIFE to unravel the mechanisms of allosteric modulation in GPCR dynamics is essential for advancing the development of novel, specific, and highly selective therapeutics.

Another significant gap in our understanding of allosteric signaling processes at the single-molecule level arises from the fact that all GPCR studies to date have utilized receptors extracted from cell membranes with the aid of detergents (Gregorio et al. 2017; Lamichhane et al. 2015; Vafabakhsh et al. 2015; Zhao et al. 2024). Detergents disrupt the native lipid-bilayer environment of membrane proteins (Bordag & Keller 2010), resulting in non-native environments that can alter their conformational landscape depending on micelle composition (Cao et al. 2021). MSP nanodiscs, which are popularly used to stabilize GPCRs into nanodiscs (Lamichhane et al. 2015, 2020; Maslov et al. 2023) cannot retain their nativeness as well. This is because they require initial detergent solubilization of the receptors, which destroys the native lipid bilayer, and reconstitution of same into synthetic lipids, which cannot accurately mimic native lipids.

To gain a comprehensive understanding of the mechanistic processes underlying GPCR signaling, it is crucial to preserve lipid-bilayer environment that closely mimic native conditions. This can be achieved in in vitro studies using native nanodiscs that retain the lipid-bilayer architecture of the parent membrane and even its local lipid composition during extraction. Such native nanodiscs can be formed using certain amphiphilic copolymers—like styrene-*co*-maleic acid (SMA) (Dörr et al. 2016; Grime et al. 2021; Jamshad et al. 2015; Logez et al. 2016) and diisobutylene-*alt*-maleic acid (DIBMA) (Grime et al. 2021; Harwood et al. 2021; Oluwole et al. 2017). New-generation copolymers, such as Sulfo-DIBMA, which has been used to extract a GPCR synthesized in a cell-free manner directly into native nanodiscs with retained ligand-binding properties (Glueck et al. 2022), Glyco-DIBMA (Danielczak et al. 2022), and poly(styrene-*co*-maleimide) (SMI) (Grime et al. 2021; Hall et al. 2018) as well as small-molecule amphiphiles like dodecyl diglucoside (DDDG) (Guillet et al. 2019; Mahler et al. 2021) have also been developed for GPCR studies. These native nanodiscs do not destroy the phospholipids’ bilayer architecture around membrane proteins, do not require the introduction of artificial lipids unlike MSPs, are stable at high temperatures, and maintain a nanoscale morphology (Danielczak et al. 2022; Dörr et al. 2016; Glueck et al. 2022; Oluwole et al. 2017). Their nanoscale morphology makes them particularly promising systems for structural elucidation of GPCRs by cryo-EM and single-molecule fluorescence studies of GPCR allostery (Ayub et al. 2024). Exploring GPCR allosteric signaling processes in native nanodiscs, therefore, offers an exciting opportunity to obtain more reliable and accurate information about these processes, deepening our understanding of these receptors and their complex mechanisms, enabled by advancements in membrane-mimetic technologies.

Although this review focusses mainly on in vitro smFRET and smPIFE experiments, live-cell single-molecule fluorescence experiments have also been adopted by many to provide crucial information for understanding GPCR signaling mechanisms. Live-cell single-molecule experiments circumvent the challenges faced during receptor extraction as it does not require receptor solubilization by membrane mimetic systems, ensuring that they are monitored in their native cellular environment. This technique has been used to study GPCR activation mechanisms (Calebiro et al. 2013; Grimes et al. 2023; Möller et al. 2020; Sungkaworn et al. 2017; Yanagawa et al. 2018; Zhou et al. 2024), receptor phosphorylation (Kawakami et al. 2022), transducer recruitment (Grimes et al. 2023; Kawakami et al. 2022; Möller et al. 2020), and receptor oligomerization (Calebiro et al. 2013; Işbilir et al. 2020; Nishiguchi et al. 2020; Zhou et al. 2024). These studies provide insights into GPCR–protein interactions, membrane organization, and allosteric signaling within the cellular context, demonstrating how in-cell single-molecule approaches complement in vitro single-molecule studies.

In conclusion, the ability of smFRET and smPIFE to directly probe the complex signaling processes of GPCRs has revolutionized our understanding of these critical membrane proteins. These techniques offer unparalleled insights into key allosteric processes, including ligand efficacy, biased signaling, and allosteric modulation, enabling the resolution of complex mechanisms that govern GPCR function. Harnessing the full potential of smFRET and smPIFE to address unanswered questions and fill existing knowledge gaps will unlock exciting new perspectives on the physiological and pharmacological roles of GPCRs, paving the way for the design of more effective and specific therapeutics.

## Data Availability

No datasets were generated or analysed during the current study.
